# The National Medical Union

**Published:** 1913-12-06

**Authors:** 


### The Hospital

The Workers' Newspaper of Administrative Medicine and Institutional Life, Administration, National Insurance and Health.

No. 1432, Vol. LV. SATURDAY, DECEMBER 6, 1913. PRICE ONE PENNY.

### THE NATIONAL MEDICAL UNION.

The result of the conference of delegates representing all the non-panel and anti-panel associations, held on Saturday, November 29, at the Waldorf Hotel, was a decision in favour of federation which is of the utmost importance to the future of panel practice. The most pressing need of these local non-panel societies, scattered and for the most part individually small, was union and centralisation. If the disasters of a year ago had not taught the medical profession the absolute necessity of organisation, it would indeed be time to despair of the future. As matters stand, the all-important first step has been taken, and the National Medical Union has been duly constituted by its constituent bodies as an association of practitioners who have nothing, directly or indirectly, to do with the panels set up by the Insurance Act. Local autonomy is preserved by all the federated local associations, which are to contribute a subscription of one guinea to the central body in respect of each of their members.

The tactical advantages—nay, the compelling and vital urgency—of this step towards union have been pointed out more than once in The Hospital during the last few weeks. The really severe strain will come when, in a year and a half's time, the valuation of the approved societies' liabilities takes place, and there is a serious risk of attempts to reduce the sums allotted for medical benefits. Before that time arrives, it is imperative that the opponents of the panel system should make up their minds what they want and how to get it. They must have a definite policy, based upon clear-cut views of the situation; they must find resourceful leaders, and must learn how to trust and follow them. There is time for all this, if action is begun at once; but there would not be time enough if the federation had been delayed much longer.

In a special supplement which we publish this week, a full account of the proceedings at Saturday's conference is given. It will be observed that the principal officers of the National Medical Union come from all parts of the kingdom; and that Scotland is as well represented, in proportion, as is England. It will also be noted that a resolution was unanimously passed to send a message of sympathy to the medical profession in Germany in its struggle against the German Insurance system. It may be inferred that the object-lesson provided by the experience of the German doctors has been taken to heart in this country, and that the danger lest a similar state of things should arise here is duly appreciated. In Germany the original sum per patient fixed for medical benefit is reported to have been reduced already by thirty-three per cent., which would mean in England that the panel doctors would receive less than six shillings per insured person per annum. It is instructive to note further that the tendency to shrinkage in payments to the German doctors is still maintained.

British practitioners on the panel ought to welcome the National Medical Union, for the stronger it becomes the more effective must be the fight which the medical profession may have to wage within the next three years. It is a momentous fact that after many hours' discussion unity should have been attained at the conference on Saturday. Unity was the keynote of every speaker. The injury done to the prestige of medical practitioners by the panel system should in consequence be effectually checked. The decisions of the conference were arrived at with practical unanimity. The National Medical Union has therefore a free hand to press forward with its work, and is entitled to have at once the support of every non-panel practitioner, who should become a member without loss of time.

It is an ungrateful and impossible business to try to forecast the future; and it may be that the approved societies, in the main, will be able to show, at the valuation of July 1915, assets exceeding their liabilities. But expert actuaries and high officials of the largest societies are pessimistic about the matter; and it is, in our opinion, as reasonably certain as anything mundane can be that if the valuations do disclose deficiencies in most of the societies, and if the present Government is still in office, a tremendous agitation will be aroused for the purpose of cutting down the pay of the doctors, in preference to reducing the other benefits as prescribed in the Act. It has to be remembered that the members of the societies possess some millions of votes, and that the Chancellor, who has openly avowed his policy of vote-catching, is a past master of that gentle art. The matter will, no doubt, interest the panel men primarily and painfully; but amid the upheaval which must follow any attempt of the kind we have indicated, a stable, well-established, well-managed association of non-panel practitioners, such as the National Medical Union promises to become, is bound to receive enormous accessions of strength. This strength will be needed, for the National Medical Union must form the principal bulwark of the medical profession against oppression and tyranny. Further, its organisation and the principles of its members will prove to be the safeguards of medicine against the derogatory tendencies of National Insurance practice and their present absolute menace to the National Health.

